# Challenges for Children 65+ Caring for Parents 90+ With Dementia During COVID-19

**DOI:** 10.1093/geroni/igab046.536

**Published:** 2021-12-17

**Authors:** Elizabeth Gallagher, Kathrin Boerner, Yijung Kim, Kyungmin Kim, Daniela Jopp

**Affiliations:** 1 University of Massachusetts Boston, Boston, MA, Massachusetts, United States; 2 University of Massachusetts Boston, Boston, Massachusetts, United States; 3 The University of Texas at Austin, Austin, Texas, United States; 4 Seoul National University, Seoul, Seoul-t'ukpyolsi, Republic of Korea; 5 University of Lausanne, Lausanne, Vaud, Switzerland

## Abstract

With the rise of the novel coronavirus, family caregivers of persons with dementia have been tasked with adapting to an entirely new caregiving landscape. Adult children caring for parents in the ‘oldest old’ age group bear an additional burden. Namely, children that are older adults themselves are navigating the joint vulnerability of both their own and their parents’ aging-related issues (e.g., health problems). The aim of this study was to explore the experiences of dementia caregivers during COVID-19 from the unique perspective of children aged 65 and older caring for parents aged 90 and older. Participants were 30 caregivers from the Boston Aging Together Study with whom we conducted in-depth interviews between March 2020 and February 2021. Thematic analysis revealed key challenges related to COVID-19. Children were worried about the prospect of their parent contracting the virus and took steps to minimize their parent’s exposure, such as discontinuing use of formal supports (e.g., home health aides) or assistance from other family and friends. Forgoing these supports often created greater responsibilities for caregivers as well as contributed to greater social isolation for both child and parent. In situations where parents resided in institutional settings, children were often unable to provide necessary help and support to parents due to restrictions. Caregivers also faced difficulties due to their parent not understanding or practicing COVID-19 regulations and in utilizing alternative means of communication with their parent (e.g., video conferencing). Supports and services should be designed in light of the unique challenges of this group.

